# Variations in IC_50_ Values with Purity of Mushroom Tyrosinase

**DOI:** 10.3390/ijms10093811

**Published:** 2009-09-02

**Authors:** Elizabeth Neeley, George Fritch, Autumn Fuller, Jordan Wolfe, Jessica Wright, William Flurkey

**Affiliations:** Department of Chemistry and Physics, Indiana State University, Terre Haute, IN 47809, USA

**Keywords:** mushroom, tyrosinase, inhibitors, IC_50_ values

## Abstract

The effects of various inhibitors on crude, commercial and partially purified commercial mushroom tyrosinase were examined by comparing IC_50_ values. Kojic acid, salicylhydroxamic acid, tropolone, methimazole, and ammonium tetrathiomolybdate had relatively similar IC_50_ values for the crude, commercial and partially purified enzyme. 4-Hexylresorcinol seemed to have a somewhat higher IC_50_ value using crude extracts, compared to commercial or purified tyrosinase. Some inhibitors (NaCl, esculetin, biphenol, phloridzin) showed variations in IC_50_ values between the enzyme samples. In contrast, hydroquinone, lysozyme, Zn^2+^, and anisaldehyde showed little or no inhibition in concentration ranges reported to be effective inhibitors. Organic solvents (DMSO and ethanol) had IC_50_ values that were similar for some of the tyrosinase samples. Depending of the source of tyrosinase and choice of inhibitor, variations in IC_50_ values were observed.

## 1. Introduction

Tyrosinase (E.C. 1.14.18.1) is a ubiquitous enzyme involved in pigmentation. It catalyzes hydroxylation of monophenols (cresolase activity) and oxidation of diphenols (catecholase activity) in the presence of molecular oxygen. Enzymes which catalyze the latter reaction are often referred to as catechol oxidases and/or polyphenol oxidases (E.C. 1.10.3.1). The distinction between polyphenoloxidases and tyrosinases is not always clear because under the right conditions many polyphenol oxidases can hydroxylate monophenols [1]. Products formed as a result of tyrosinase activity can produce precursors for skin pigmentation, defense and protective mechanisms in plants and fungi, and pigments for some flowers [2–6]. On the other hand, products from tyrosinase activity can also lead to deleterious effects such as those associated with browning reactions in fruits and vegetables and black spotting of shrimp and lobsters [7–8].

Commercial mushroom tyrosinase (MT) is often used to screen for anti-browning agents and skin whitening agents isolated from natural sources and made synthetically. As such, it serves as an *in vitro* model for the human tyrosinase in the search for decreasing skin pigmentation. Commercial MT preparations differ in tyrosinase activity, the presence of carbohydrates, organic material, and other proteins and enzymes [9–10]. All of these contaminants have the potential to affect tyrosinase activity *in vitro*, especially with regard to screening compounds for tyrosinase inhibition. Several reports have appeared that indicate commercial MT preparations contain laccase [9–12]. Laccase activity can interfere with estimations of tyrosinase activity since the enzyme can use many of the same substrates as tyrosinase. In addition, laccase contamination in commercial MT preparations, and not tyrosinase, was attributed to quinone methide formation [12]. The effects of glycosidase activities in commercial MT preparations are just as problematical. For example, esculin, a phenolic glycoside with no tyrosinase inhibitory activity, can be converted into esculetin, a phenolic tyrosinase inhibitor, by a glucosidase present in commercial MT preparations [9]. Therefore, use of tyrosinase for screening of tyrosinase inhibitors may depend on the purity of the enzyme as well as the assay conditions. It has been pointed out that MT has different enzymatic and physical properties compared to the human enzyme and extrapolation of data using MT may not be transferrable to reactions found in humans [13,14].

Over the last few years, several hundred tyrosinase inhibitors have been reported. In the majority of these examples, commercial MT was used to screen or evaluate these inhibitors. These inhibitors vary from simple compounds like NaCl and DMSO to more complex organic compounds isolated from natural sources and those compounds synthesized chemically. Reviews of MT and its inhibitors have appeared that detail properties of MT and some of the natural and synthetic inhibitors of MT [15–17].

Screening for tyrosinase inhibitors has many problems and pitfalls. If a monophenol (*i.e.,* tyrosine) is used as a substrate, there is often a lag period when monitoring the enzymatic activity. Steady state rates for determining tyrosinase activity may appear after a long time period. Compounds which act as inhibitors may extend this lag period and make determination of steady state rates more difficult and time consuming. Monitoring oxidation of a diphenol (*i.e.,* DOPA) in the presence of inhibitors is also problematical. Steady state rates are often determined from the linear portion of these curves whenever possible. The determination of steady state rates can problematical in the presence of tyrosinase inhibitors because the absorbance *vs.* time curve shapes can vary with the concentration of inhibitor and the type of inhibitor. This makes estimations of steady state rates more difficult since the linear portion of the curve can change in duration and when it is first observable. This also implies that end point assays, absorbance measurements at two different time points, may not be reliable indicators of steady state rates with regard to MT.

Because the majority of reports use commercial MT as a source of tyrosinase, we examined whether the purity of the enzyme could affect estimations of IC_50_ values, a parameter often used to indicate the potency of a tyrosinase inhibitor. We chose 18 reported tyrosinase inhibitors to test their effect on crude, commercial, and purified MT. These inhibitors were chosen based on their availability from commercial sources and our own interest in them.

## 2. Results and Discussion

IC_50_ values for 18 inhibitors of MT were determined using a crude MT extract, commercial MT, or a purified MT sample. Commercial and purified MT isolated from commercial preparations contained no latent tyrosinase. Crude extracts of MT appeared to contain latent tyrosinase and assays were conducted in the presence of 0.1% SDS to account for latent and active enzyme present (data not shown). We arranged these inhibitors into groups based on IC_50_ value similarities between the different tyrosinase samples and to IC_50_ values for commercial and/or purified MT reported in the literature (Table 1 and references therein [18–31]).

The first group of inhibitors (kojic acid, SHAM, 4 HR, tropolone, methimazole, ATTM) appeared to have similar IC_50_ values for both the commercial and purified MT (Table 1). These IC_50_ values closely resembled or were within ranges to those IC_50_ values reported in the literature. Using crude MT, IC_50_ values for these inhibitors, with perhaps the exception of 4 HR, were also similar to those obtained using commercial or purified MT. 4-Substituted resorcinols, along with tropolone, kojic acid, and mimosine referenced therein [32], were reported to be slow binding competitive inhibitors of tyrosinase and showed biphasic absorbance *vs.* time curves. We also observed biphasic absorbance *vs.* time curves for these inhibitors and only used initial rates for determination of their IC_50_ values. Some investigators use end point assays, measuring absorbencies at two different time points for rate estimations, to determine IC_50_ values for tyrosinase inhibitors. For slow binding inhibitors, and other compounds not yet identified as slow binding inhibitors, use of end point assays may lead to IC_50_ values that are not similar to that those measured under initial rate conditions or constant rate conditions. Park *et al.* [23] reported inhibition of tyrosinase by ATTM. Their absorbance *vs.* time curves also showed a biphasic response, which suggests this compound may also be a slow binding inhibitor. We also observed these biphasic curves and inhibition by ATTM below 10–20 μM. However, at higher concentrations of ATTM we observed absorbance *vs.* time curves that showed an initial decrease in absorbance that eventually “leveled out” and later began a gradual increase in absorbance depending on the concentration of ATTM. Thus, it was difficult to determine rates at greater than 10 μM ATTM.

**Figure 1.**
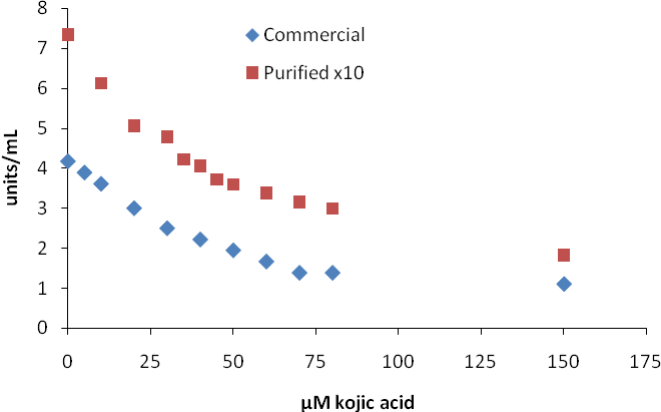
Inhibition of mushroom tyrosinase by kojic acid. Assays were carried out as described in the Experimental section.

Many investigators use kojic acid as a reference inhibitor, and it is surprising that IC_50_ values for kojic acid vary over such a wide range (Table 1, [18]). Our IC_50_ values are within the lower end of these ranges. Figure 1 shows a plot of MT activity *vs.* kojic acid concentration for commercial and purified MT used to determine an IC_50_ value. We show these two curves because most IC_50_ values are reported using the commercial MT and sometimes using a purified MT. Even though the commercial and purified tyrosinase showed different enzyme activity in the absence of kojic acid, the IC_50_ values and inhibition curves were similar. In general, for inhibitors in group 1 it appears that the purity of tyrosinase does not have a significant effect on IC_50_ values.

The second group of inhibitors (NaCl, esculetin, biphenol, phloridzin) seemed to show a few differences in IC_50_ values between the crude, commercial and purified enzyme (Table 1). For the most part, this group of inhibitors also appeared to have much larger IC_50_ values than those reported in Table 1. For example, NaCl and esculetin seemed to show an increase in IC_50_ values from crude to commercial to purified MT. In contrast, biphenol did not show quite as large a variation in IC_50_ values among the tyrosinase samples, although the IC_50_ for biphenol was much smaller for commercial MT. Except for phloridzin, the IC_50_ values for NaCl, esculetin and biphenol seemed to be larger for the purified enzyme compared to the commercial or crude enzyme in this group of inhibitors.

**Figure 2.**
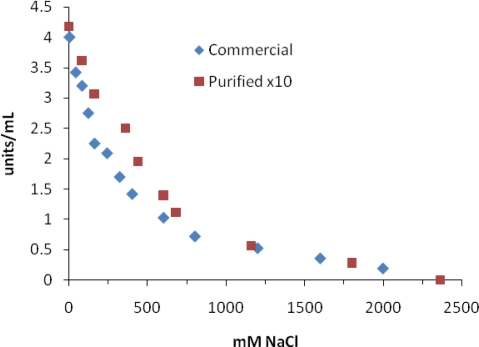
Inhibition of mushroom tyrosinase by NaCl. Assays were carried out as described in the Experimental section.

Figure 2 shows the plot of MT activity *vs.* NaCl concentration using the commercial and purified MT. These IC_50_ values for NaCl were much higher than that reported by Park *et al.* [24], who suggested that Cl^−^ was responsible for inhibition and not Na^+^. They also used DOPA as a substrate for tyrosinase. We have no reasonable explanation for the difference in our results *vs.* those by Park *et al.* [24] except for the source of commercial tyrosinase used in the two studies. However, even our purified MT showed a much higher IC_50_ value than the commercial MT.

**Figure 3.**
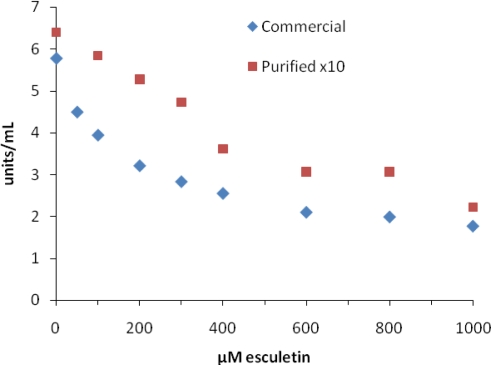
Inhibition of mushroom tyrosinase by esculetin. Assays were carried out as described in the Experimental section.

Figure 3 shows a plot of activity of MT *vs.* esculetin concentration for commercial and purified MT. IC_50_ values for esculetin were much higher than that reported by Masamoto [25], although Flurkey *et al.* [10] did report a comparable IC_50_ value for esculetin using commercial MT. Even though esculetin has been reported to be an inhibitor, Munoz-Munoz *et al.* [33] have shown that esculetin can be used as an alternative substrate for MT and peroxidase using a chronometric assay. Sollai *et al.* [34] has also shown that esculetin can be oxidized by MT, plant polyphenol oxidases, and laccase although at slow rates. Thus, apparent inhibition by esculetin could be explained by competition for two different substrates. However, it is still hard to reconcile the differences in IC_50_ values between the MT samples we tested, especially the 685 μM IC_50_ value for purified MT.

Kim *et al.* [26] obtained an IC_50_ value for biphenol of approximately 2 μM using tyrosine as a substrate instead of DOPA, although they monitored dopachrome formation from tyrosine. Using DOPA as a substrate, we observed a large variation in IC_50_ values for biphenol between crude and purified MT *vs.* commercial MT. In addition, we observed increases in tyrosinase activity at concentrations above 20 μM for commercial MT and 100 μM biphenol for purified MT (data not shown). Using crude tyrosinase extracts, there was a 50% decrease in enzyme activity from 0 to 50 μM biphenol but no further inhibition was observed up to 500 μM biphenol (data not shown).

In general, it was difficult to discern a consistent pattern in IC_50_ values in group 2 with regard to the purity of tyrosinase. It is possible that some of these inhibitors could be affected by material in crude extracts and in commercial samples of MT differently or behaved differently with a more purified source of enzyme.

**Figure 4.**
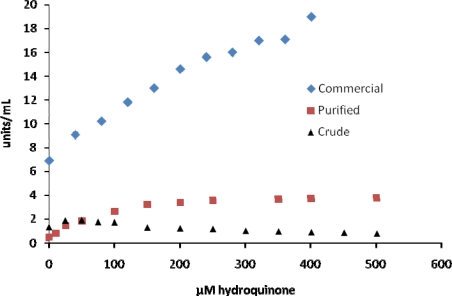
Inhibition of mushroom tyrosinase by hydroquinone. Assays were carried out as described in the Experimental section.

The third group of inhibitors (hydroquinone, lysozyme, ZnSO_4_, anisaldehyde) tested seemed to show little or no inhibition of tyrosinase or required concentrations to reach 50% inhibition that were much different than those reported by other investigators (Table 1). This was true for all of the different tyrosinase samples tested. For example, using DOPA as a substrate we found that hydroquinone increased reactions rates at lower concentrations of hydroquinone and caused inhibition at higher concentrations for some of the enzyme samples (Figure 4). Although hydroquinone has been reported to be an inhibitor of MT, Kasraee [35] and Passi and Nazzaro-Porro [36] have reported that hydroquinone can act as a substrate for MT in which the oxidation product of hydroquinone, parabenzoquinone, can oxidize DOPA. Enhancement in our observed rates at lower concentrations of hydroquinone could be a result of hydroquinone coupled oxidation of DOPA as well as enzyme catalyzed oxidation of DOPA. This apparent activation varied between 20–500 μM hydroquinone for the different MT samples (Figure 4). The crude tyrosinase showed a decrease in activity beyond 100 μM hydroquinone, while the commercial enzyme showed increased activity up to 400 μM hydroquinone. In contrast, the purified enzyme showed an increase in activity with increasing hydroquinone up to 200 μM and then activity seemed to remain relatively constant up to 500 μM hydroquinone.

For lysozyme, we could not add enough inhibitor into the assay to determine an IC_50_ value and observed less than 50% inhibition, even with 10–20 mg/mL (700–1,400 μM) lysozyme in the assay. This is in contrast to Li *et al.* [28], who found that lysozyme inhibited MT with an IC_50_ value of 0.32 μM (4.6 μg/mL). They also suggested that inhibition was dependent upon the time of pre-incubation of lysozyme with tyrosinase. For example, Li *et al.* [28] observed 8% inhibition on mixing MT with lysozyme just before assaying but found 30% inhibition if MT and lysozyme were incubated for one hour before assaying. We found 8–25% inhibition when large amounts of lysozyme (10–20 mg/mL) were mixed with MT just before assaying, but did not attempt to incubate MT and lysozyme for various times to increase the percent inhibition. We also tried three different sources of lysozyme with the similar results (data not shown).

Effects of Zn^2+^ and anisaldehyde on mushroom tyrosinase were examined. In monitoring the oxidation of DOPA to dopachrome, we found no inhibition of MT by Zn^2+^, even at concentrations up to 1 mM (Table 1). This is in contrast to that reported by Han *et al.* [29]. However, they used a much different assay for tyrosinase that coupled DOPA oxidation to a change in the coloration of thymol blue as a result of protonation. Since our assays monitor DOPA oxidation directly, we can only surmise that Zn^2+^ may cause some sort of interference in the coupled assay reported by Han *et al.* [29]. We were able to determine IC_50_ values for anisaldehyde using the crude, commercial and purified MT (Table 1). These values ranged from 1 to 1.8 mM between the mushroom samples but were much larger that that reported by Ha *et al.* [30]. Although we did not check for the presence of anisic acid in the anisaldehyde, anisic acid has been shown to be a MT inhibitor with an IC_50_ value of 680 μM [37]. In general, the IC_50_ values in this third group of inhibitors were much different than that reported by citations listed in Table 1 even though the crude, commercial and purified MT appeared to behave in a similar manner with these inhibitors.

The fourth group of potential MT inhibitors (DMSO, dimethyl sulfide, dimethyl sulfone, ethanol) comprised compounds that are commonly used to dissolve MT inhibitors and/or may be potential contaminants in DMSO preparations (Table 1). IC_50_ values for ethanol were similar for all MT samples tested. Little to no inhibition was observed for dimethyl sulfide up to 500 μM using any of the MT samples tested. Perez-Gilabert and Garcia-Carmona reported that dimethyl sulfide is a slow binding inhibitor of tyrosinase, although they did not report IC_50_ values [38]. We also observed relatively little inhibition of tyrosinase up to 2 mM dimethyl sulfone.

Inhibition by DMSO did not appear to be much different for crude or commercial MT even though the IC_50_ values were larger that using the purified MT. Our IC_50_ values for DMSO using commercial and purified MT were somewhat lower than that reported by Chen *et al.* [31]. We did find that inhibition by DMSO might be dependent on the source and/or purity of the DMSO used (data not shown). We also observed that high concentrations of DMSO caused a precipitate to form in the assay solution, presumably inorganic compounds that are less soluble as the DMSO increased in concentration. DMSO has also been shown to cause protein precipitation and denaturation by Arakawa *et al.* [39] at higher DMSO concentrations. In addition, Tjernberg *et al.* [40] reported that even low concentrations of DMSO (< 5 %) can destabilize proteins. These effects may have to be considered when examining inhibitory effects of organic solvents or inhibitors dissolved in organic solvents, like DMSO, on tyrosinase activity. In general, and with the exception of DMSO, the fourth group of potential tyrosinase inhibitors displayed similar IC_50_ values for the different sources of tyrosinase.

## 3. Experimental Section

### 3.1. Materials

Commercial mushroom tyrosinase was purchased from Worthington Biochemical Corp. (Lakewood, NJ, USA). Substrates and inhibitors were obtained from Sigma-Aldrich (St. Louis, MO, USA). Anisaldehyde was obtained form J.T. Baker Chemical Co. (Phillipsburg, NJ, USA). Dimethyl sulfoxide (DMSO) was reagent grade from Sigma-Aldrich (St. Louis, MO, USA).

### 3.2. Methods

#### 3.2.1. Crude Extract Tyrosinase Preparation

White button mushrooms were purchased from local stores and blended in 100 mM sodium phosphate (pH 6.0) containing 1 mM EDTA, 1 mM PMSF, 5 mM ascorbic acid and 1 M sucrose. The homogenates were filtered and stored at −15 °C in small aliquots. Commercial mushroom tyrosinase. Mushroom tyrosinase was dissolved in the above buffer without ascorbic acid at 2 mg dry weight/mL and stored at 5 °C in small aliquots.

#### 3.2.2. Enzyme Purification

Tyrosinase was partially purified from commercial mushroom tyrosinase preparations using ammonium sulfate precipitation (35–60%), ion exchange on DEAE Sepharose CL-6B, hydrophobic interaction on Phenyl Sepharose in 1 M NaCl, and adsorption on hydroxyapatite [41,42].

#### 3.2.3. Enzyme Assays

Each one mL assay contained a final concentration of 100 mM sodium phosphate (pH 6.5) and 2 mM DOPA. Crude, commercial or partially purified tyrosinase was added to initiate the reaction. Oxidation of DOPA into dopachrome was monitored at 475 nm. One unit of activity was defined as 1 μmole dopachrome formed per min per ml enzyme using an extinction coefficient of 3600 M^−1^ cm^−1^. Rates were determined from the linear portion of the absorbance *vs.* time curves. Crude enzyme was use at a concentration of 0.1 units (8.4 μg protein) per mL in the assay and assayed in the presence of 0.1% SDS. Commercial tyrosinase was used at a concentration of 0.038 units (3.3 μg protein) per mL in the assay. The purified enzyme was used at a concentration of 0.0067 units (0.58 μg protein) per mL in the assay.

#### 3.2.4. IC_50_ Determinations

Various amounts of inhibitors were added into assays to determine a range of inhibitors needed to encompass the IC_50_ value. Then assays were repeated within this range of inhibitor concentrations. Rates were determined as above. If any inhibitor caused a significant change in the shape of the A *vs.* time curve, rates were calculated from steady state rate regions (linear portions) in the curves. The concentration of inhibitor needed to inhibit 50% of the tyrosinase activity was extrapolated from % activity *vs.* [inhibitor] curves. For some inhibitors, we also used Statistical Data Analysis Software (SDAS, version 2008A) using an exponential equation to obtain a best fit curve and then to calculate IC_50_ values. For the crude and commercial MT, experiments were repeated at least twice and the average IC_50_ value reported. Because of the limited amount of purified tyrosinase, not all experiments could be repeated twice. In experiments requiring DMSO to dissolve the inhibitor, control assays contained the assay mixture with DMSO but minus inhibitor. Final DMSO concentrations were 5% or less in the assays.

## 4. Conclusions

IC_50_ values for tyrosinase inhibitors can vary for a number of reasons, including choice of assay conditions, rate determinations, and solvent considerations. Therefore, it is sometimes difficult to reproduce data reported in the literature. Our results seem to indicate the IC_50_ values can also vary depending on the purity of the tyrosinase. However, this observation also seems to be related to the type of inhibitor used. Crude MT extracts and commercial MT sources contain a variety of proteins, enzymes, carbohydrate, and organic compounds. Any one of these substances can interfere with IC_50_ value estimations through chemical or enzymatic reactions. We suggest that MT preparations need to be partially purified to eliminate these potential problems in IC_50_ value determinations.

## Figures and Tables

**Table 1.** IC_50_ values for mushroom tyrosinase inhibitors.

**Inhibitor**	**Crude MT μM**	**Commer. MT μM**	**Purified MT μM**	**Reported IC_50_ Values μM**
kojic acid	36	47	45	10–300 [10,18]
SHAM	0.24	0.4	0.45	0.45 [19], 0.8, 1.2 [10]
4-HR	5.8	1.5	1.2	0.85 [20], 2.5, 18 [10]
tropolone	0.39	1.2	0.95	0.4 [21], 1.3, 1.7 [10]
methimazole	46	55	50	200 [22], 40, 47 [10]
ATTM	3.4	3.3	2	2 [23], 7.5, 28 [10]
NaCl	65 mM	270 mM	360 mM	25 mM [24]
esculetin*	70	280	685	43 [25], 210, 225 [10]
biphenol*	50^a^	10^b^	67^c^	1.9 (tyrosine) [26]
phloridzin*	625	1740	1400	110 [27]
hydroquinone	activates^d^	activates^e^	activates^f^	26 [18]
lysozyme	>10 mg/mL^g^	>20 mg/mL^h^	>20 mg/mL^i^	0.32 μM [28]
ZnSO4	none@1 mM	none@1 mM	none@1 mM	50 [29]
anisaldehyde*	1 mM	1.4 mM	1.8 mM^j^	160 [30]
DMSO	2 M	1.75 M	1.4 M	2.45 M [31]
dimethyl sulfide	NI to 500 μM	NI to 500 μM	NI to 500 μM	
dimethyl sulfone	NI to 2 mM	NI to 2 mM	NI to 2 mM	
ethanol	2.3 M	2.1 M	1.8 M	

Assays were carried out as described in the experimental section.

a ) 50% inhibition at 50 μM, no decrease in activity beyond 100 μM;

b ) 50% inhibition at 10 μM, increases in activity beyond 10 μM;

c ) 50% inhibition at 67 μM, increases in activity beyond 100 μM;

d ) activated up to 100 μM, 50% inhibition at 800 μM;

e ) activated up to 400 μM;

f ) activated up to 500 μM;

g ) 25% inhibition at 10 mg/mL;

h ) 18% inhibition at 20 mg/mL;

i ) 8% inhibition at 20 mg/mL;

j ) 50% inhibition at 1.8 mM, remained at 60% from 2–5 mM;

* - indicates compounds dissolved in DMSO; NI-no inhibition.

## Abbreviations:

ATTM: ammonium tetrathiomolybdate
biphenol: 4,4’-dihydroxy-biphenyl
DOPA: dihydroxyphenylalanine
DMSO: dimethyl sulfoxide
EDTA: ethylenediaminetetraacetic acid
4HR: 4-hexylresorcinol
MT: mushroom tyrosinase
PMSF: phenylmethylsulfonyl fluoride
SDS: sodium dodecylsulfate
SHAM: salicyl-hydroxamic acid
